# Correlation Between Different Sagittal Parameters in Patients With Degenerative Kyphosis

**DOI:** 10.3389/fnmol.2022.847857

**Published:** 2022-03-22

**Authors:** Yan Liang, Shuai Xu, Chen Guo, Keya Mao, Haiying Liu

**Affiliations:** ^1^Spinal Surgery, Peking University People’s Hospital, Beijing, China; ^2^The Chinese PLA General Hospital (301 Hospital), Beijing, China

**Keywords:** degenerative kyphosis, lumbar lordosis, thoracolumbar kyphosis, pelvic incidence, pelvic tilt, sagittal balance

## Abstract

**Objective:**

To explore the relationship between different sagittal parameters and identify the fitting formula of spino-pelvic parameters in patients with degenerative kyphosis (DK).

**Summary of Background:**

Sagittal balance is increasingly recognized as a predictor of clinical outcomes in patients with DK, while the relationship between different sagittal parameters in patients with DK remains unidentified.

**Methods:**

A retrospective study with 279 participants was conducted. There were 168 DK patients which were divided into a sagittal balance group (SB:52 cases) and sagittal imbalance (SIB:116 cases). Radiographic measurements included thoracolumbar kyphosis (TLK), lumbar lordosis (LL), thoracic kyphosis (TK), pelvic incidence (PI), and pelvic tilt (PT). The correlations were analyzed between different sagittal parameters.

**Results:**

There were significant differences between the SB and SIB groups in terms of TLK, LL, PI-LL, PT, SVA, sacral slope (SS), and TK. For patients with DK, the LL was correlated with PT and TK. The linear regression was LL = 22.76−0.28 × PT + 0.62 × TK. In the SB group, TK was the influencing factor for LL and the linear regression analysis showed that LL = 33.57 + 0.33 × TK. While in the SIB group, PT and TK were in synergistic effect with PI-LL, the linear regression analysis showed that LL = 22.76−0.28 × PT + 0.62 × TK.

**Conclusion:**

From the present study, we can see that LL has a significant correlation with PT and TK in patients with DK, while in SB, the LL was only correlated with TK. Therefore, the correction of LL in a different group should be calculated to avoid the incidence of proximal junction kyphosis (PJK).

## Introduction

The typical sagittal sequence of the spine and the balance status of the lumbar-pelvis are the main factors for spinal kinematics and energy expenditure. In a normal population, the structure of the spine is closely related to biomechanics and forms a unique “S-shaped” bend through bone adjustment (Roussouly and Pinheiro-Franco, 2011; Diebo et al., 2015). Lumbar kyphosis maintains a stable center of gravity in the area between the feet, maximizing energy efficiency while minimizing the impact of gravity on the joints, muscles, and ligaments with the “energy cone” (Le Huec et al., 2019). The ideal spino-pelvic sagittal sequence describes the ideal dynamic matching of bones in the sagittal position due to the interaction among various segments, while the focal disorders or breaking of the balance will cause sagittal deformity and a compensatory mechanism by other parts in order to minimize the energy cost (Liu et al., 2020).

With the aging of the population, the number of patients with degenerative kyphosis (DK) is gradually increasing by the spinal degeneration of vertebral deformation, facet disorders, disc aging, as well as osteophyte formation (Lee et al., 2011). DK is mainly characterized by the decrease of lumbar lordosis (LL) and/or the increase of thoracolumbar kyphosis (TLK) with enlarged prevalence in the elderly accompanied with or without neurological deficit. In cases of DK with either condition, the proximal and distal levels of the spine and pelvis are compensated accordingly to maintain a healthy state of balance. Once the compensatory capacity is overloaded, it leads to an unbalanced state (Schwab et al., 2012, 2013; Wu et al., 2014).

The spino-pelvic alignment has been quantified by a series of parameters with uniformed theory. Dubousset et al. (2005) proposed that the pelvis is the cornerstone of the sagittal sequence of the spine, and the pelvic tilt (PT) established a possible compensatory mechanism for spinal imbalance. Ginette Duval-Beaupere defined the geometric meanings of pelvic incidence (PI), PT, and sacral slope (SS): PI = PT + SS (Duval-Beaupere et al., 1992), and the pelvic incidence–lumbar lordosis (PI–LL) <10° threshold was determined as the standard for spinal-pelvic sagittal fitting (Schwab et al., 2010), which linked the relationship between lumbo-pelvic matching.

Many studies have specifically discussed the correlation among sagittal spinal parameters. Kim pointed out that the loss of LL is the initial of spino-pelvic malalignment and the key factor of compensation (Kim et al., 2014). Lafage et al. (2016) proved that the compensatory mechanism of the spine usually starts from the flexible segments with a broad range of motion and gradually extends distally. The body compensates by moving the center of gravity back and forth, beginning with the traction of thoracic vertebrae, then regulated by pelvic rotation and hip-knee flexion. Schwab et al. (2013) put forward the correction formula LL = (PI + TK)/2 + 10 in adult spinal deformity, which skillfully correlates more parameters to fit the real sagittal sequence. However, the relationship among sagittal spino-pelvic parameters and the influence of spino-pelvis matching is seldom featured in patients with DK. Therefore, by enrolling the patients with DK, the purpose of this study was to explore the relationship among sagittal spino-pelvic parameters and identify the fitting formula of spino-pelvic parameters by the lumbo-pelvic balance status.

## Materials and Methods

### Participants

**FIGURE 1:**
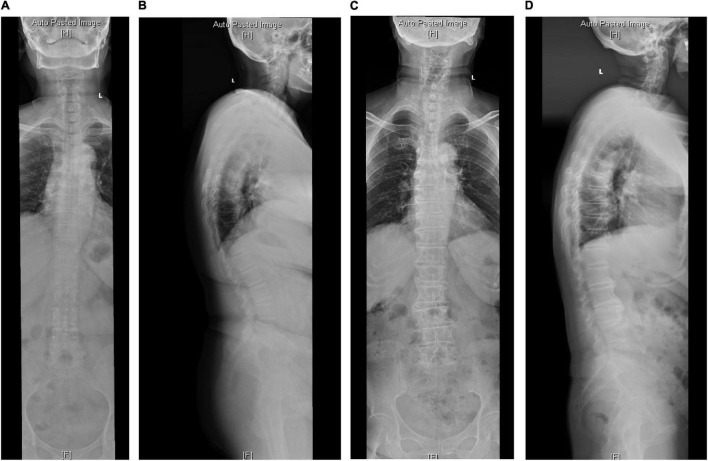
The radiological parameters measured on standard anterior-posterior and lateral whole spine X-ray of cases from the SB group. **(A,B)** In a 70-year-old man, TK = 58, TLK = 26.1, LL = 56.1, PI = 51, PT = 20.4. **(C,D)** In a 68-year-old woman, TK = 29.4, TLK = 15.2, LL = 35.3, PI = 44.7, PT = 21.

**FIGURE 2:**
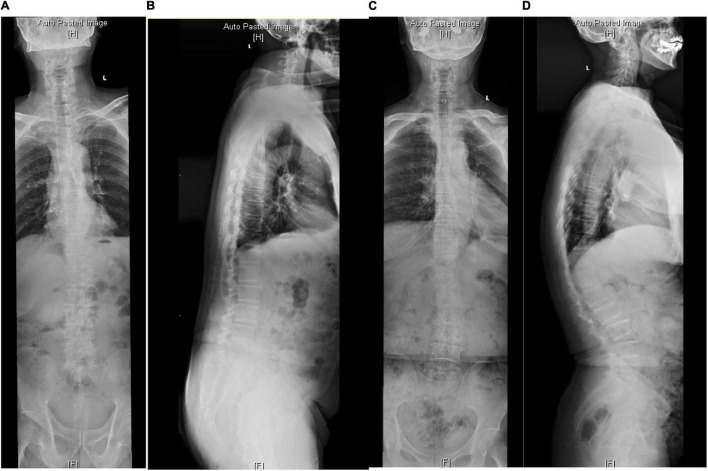
The radiological parameters measured on standard anterior-posterior and lateral whole spine X-ray of cases from the SIB group. **(A,B)** In a 68-year-old woman, TK = 16.4, TLK = 1.1, LL = 19.5, PI = 63.4, PT = 39.9. **(C,D)** In a 72-year-old man, TK = 40.9, TLK = 40, LL = 72.2, PI = 51.7, PT = 3.7.

A single-center retrospective study was performed in our institution from June 2016 to June 2020. The patients with DK of the whole spine were included, composed of the degenerative thoracolumbar kyphosis (DTLK) or loss of LL. According to the viewpoints of Schwab et al. (2007), PI-LL was a key parameter for quantifying sagittal balance, where a matched condition was PI-LL ≤ ± 10°. Hence, the series was divided into 2 groups, sagittal balance (SB) group: | PI-LL| ≤ 10° (Figure 1) and sagittal imbalance (SIB) group: | PI-LL| > 10° (Figure 2; Lafage et al., 2017). The participants from the two groups were selected with a matching of 1:2 by propensity score matching. The protocol was approved by the ethics committee and all participants have signed the consent forms.

The inclusion criteria were (1) patients with TLK, loss of LL, or both caused by degeneration; (2) whole-spine X-ray and lumbar spine X-ray were completely obtained; and (3) an age of more than 50 years for all participants. The exclusion criteria were (1) patients sustaining coronary imbalance; (2) blurred images of radiographs for measurement; (3) patients with idiopathic scoliosis or other types of deformities; (4) patients with spine tumors, infections, or spondylolisthesis; and (5) patients who had undergone lumbar spine surgery before.

### Radiological Parameters

Thoracolumbar kyphosis was the angle between the upper endplate of T10 and the lower endplate of L2 and DTLK was TLK ≥15° with degenerative factors; LL was the angle between the upper endplate of L1 and the upper endplate of S1 and the loss of LL was LL ≤25°. TK was the angle between the upper endplate of T5 and the upper endplate of T12. The spino-pelvic parameters contained PI-LL and PT in this study. PT was the angle between the plumb line and the center of the femoral head to the midpoint of the upper endplate of S1. PI was defined as the vertical line passing through the midpoint of the upper endplate on S1, then the second line connecting the midpoint of the upper endplate on S1 and the femoral head and the angle between the second line and vertical line. Sagittal vertical axis (SVA) was the axis. All parameters were independently measured by two reviewers, and the mean value was adopted.

### Statistical Analysis

The dichotomy between SB and SIB groups were analyzed by χ^2^ test. An independent sample *t*-test was used for inter-group measurement data. Pearson correlation analysis was used to determine the relationship between LL, PI-LL, PT, and TK. Multiple linear regression analysis was for determining the influencing factors of PI-LL and LL. SPSS 22.0 (IBMC, Armonk, NY, United States) the software for statistical analysis, and *P* < 0.05 was statistically significant.

## Results

### Patient Clinical Characteristics

A total of 168 patients was included with 52 cases in the SB group and 116 cases in the SIB group. There were no differences between the subgroups in terms of gender (*P* = 0.555), age (*P* = 0.686), and BMI (*P* = 0.278). Thirty-seven cases both had TLK and loss of LL, and TLK was the majority (94.3%) in the SB group in contrast with the group with unmatched PI-LL (*P* < 0.001). There was a significant difference between the SB and SIB groups in terms of TLK, LL, PI-LL, PT, SVA, SS, and TK (*P* < 0.05) (Tables 1, 2).

**TABLE 1:** Basic information in matched and unmatched groups.

	SB	SIB	P
Gender, M:F	19:33	37:79	0.555
Age, y	67.3 ± 8.8	66.6 ± 9.9	0.686
BMI, kg/m^2^	25.3 ± 3.4	26.0 ± 4.0	0.278
No. of increased TLK	50	78	<0.001
No. of decreased LL	3	74	

*BMI, body mass index; TLK, thoracolumbar kyphosis; LL, lumbar lordosis.*

**TABLE 2:** Spino-pelvic parameters in matched and unmatched groups.

	SB	SIB	P
TLK,°	25.4 ± 12.3	18.4 ± 15.2	0.004
LL,°	45.8 ± 11.9	23.4 ± 20.8	<0.001
PI,°	46.5 ± 10.4	44.6 ± 12.1	0.325
PI-LL,°	0.7 ± 5.2	21.2 ± 18.1	<0.001
PT,°	16.1 ± 6.6	23.5 ± 10.7	<0.001
SVA, mm	25.7 ± 27.2	56.4 ± 50.6	<0.001
TK,°	34.9 ± 13.8	21.2 ± 15.9	<0.001
SS,°	30.5 ± 10.0	21.1 ± 11.0	<0.001

*TLK, thoracolumbar kyphosis; LL, lumbar lordosis; PI, pelvic incidence; PT, pelvic tilt; SVA, sagittal vertical axis; TK, thoracic kyphosis; SS, sacral slope.*

### Pearson Correlation Analysis

In the SB group, PI-LL was negatively related to LL (*r* = −0.492, *P* < 0.001), and TK was positively related to PT (*r* = 0.440, *P* = 0.02) and LL (*r* = 0.370, *P* = 0.008). In the SIB group, PI-LL was positively correlated to PT while negatively related to TK and LL (*P* < 0.01). LL was correlated to PT (*P* = 0.037) and TK (*P* < 0.001) (Table 3 and Figures 1, 2).

**TABLE 3:** Correlation analysis among PI-LL, PT, and TK in matched and unmatched groups.

		PI-LL		PT		TK	
		r	P	r	P	r	P
SB	PT	0.244	0.085				
	TK	−0.258	0.071	0.440	0.002		
	LL	−0.492	<0.001	0.222	0.117	0.370	0.008
SIB	PT	0.699	<0.001				
	TK	−0.522	<0.001	−0.073	0.471		
	LL	−0.801	<0.001	−0.303	0.001	0.634	<0.001
Total	PT	0.700	<0.001				
	TK	−0.564	<0.001	−0.094	0.255		
	LL	−0.827	<0.001	−0.357	0.000	0.650	<0.001

*PI, pelvic incidence; LL, lumbar lordosis; PT, pelvic tilt; TK, thoracic kyphosis.*

### Multiple Linear Regression Analysis

In the SB group, the linear regression analysis showed that TK was the influencing factor for PI-LL and LL (PI-LL = 0.47 × PT−0.46 × TK and LL = 33.57 + 0.33 × TK). While in the SIB group, PI-LL, PT, and TK were in synergistic effect with PI-LL = 6.81 + 0.66 × PT−0.47 × TK and LL = 18.2–0.26 × PT + 0.62 × TK. When integrating the SB and SIB groups, PI-LL, PT, and TK were shown to be synergistic parameters regardless of being in a matched and unmatched group: PI-LL = 4.92 + 0.64 × PT−0.50 × TK and LL = 22.76−0.28 × PT + 0.62 × TK (Table 4).

**TABLE 4:** Multiple linear regression analysis on LL in matched and unmatched groups.

	Coefficient	Unstandardized		Standardized	T	P
		B	SE	Beta		
SB	(constant)	33.565	5.091		6.593	<0.001
	PT	0.133	0.279	0.073	0.478	0.635
	TK	0.291	0.133	0.333	2.186	0.034
SIB	(constant)	18.200	4.147		4.389	<0.001
	PT	−0.476	0.136	−0.261	−3.508	0.001
	TK	0.801	0.097	0.615	8.262	<0.001
Total	(constant)	22.756	3.444		6.607	<0.001
	PT	−0.563	0.117	−0.284	−4.817	<0.001
	TK	0.789	0.075	0.623	10.588	<0.001

*LL, lumbar lordosis; PT, pelvic tilt; TK, thoracic kyphosis; SE, standard error.*

## Discussion

The sagittal spino-pelvic alignment has an important role in maintaining the normal function of the spine and provides explicit guidance for surgical reconstruction. The degenerative progression of sagittal deformity of the spine consumes a lot of energy for patients and may accelerate the progress of other spinal degenerative diseases (Kim et al., 2014; Lee et al., 2017). Therefore, many studies have attempted to explore and quantify the correlation between spino-pelvic parameters to provide clinical evidence. Schwab et al. (2010) quantified the relationship between the pelvis and LL by PI minus LL, and determined the PI-LL <10° as the standard threshold of spino-pelvic match (Barrey et al., 2007). Then, the team proposed that LL exceeding PI is relatively appropriate in patients with low PI according to the clinical practice, conversely, LL is less than PI; and high TK also need a larger LL than the theoretical value to compensate for thoracic kyphosis (TK), that is, LL = (PI + TK)/2 (Le Huec et al., 2015a). A later study found that there is a correlation between parameters and age. In the middle-aged population, the normal range of parameters is different from that of young people. Lafage et al. (2017) believe that elderly patients have more compensation, more serious loss of LL and more anteversion, so they put forward an age-related correction formula. Zhou et al. (2019) studied the sagittal shape and sequence matching relationship of spino-pelvis in normal middle-aged population, and pointed out that there were great differences in pelvic parameters between China and foreign countries: LL = 0.6PI + 0.4TK + 10.

The normal sequence of lumbar vertebrae is the basis for the study of lumbar development and degenerative diseases and the premise of reconstruction surgery. Mangione et al. (1997) believe that to maintain a balanced posture with minimum energy consumption, the best sequence of the lumbar spine depends on the PI, while the definition of PT establishes a possible compensatory mechanism for spinal imbalance and quantifies the pelvic rotation around the femoral head. The sagittal alignment was a morphology parameter that correlated with anatomy, and the balance was a dynamic characteristic (Moal et al., 2015). Spinal degeneration can involve any segment, and the loss of LL is the main cause of sagittal sequence loss. PT, as a sensitive parameter of spino-pelvic mismatch, is the main compensatory mechanism. The continuous loss of lumbar kyphosis is usually accompanied by an increase in pelvic rotation and TLK, resulting in DK. For such patients, the compensation mechanism usually starts from the flexible segments with a large range of movement (Kim et al., 2014). With the pelvis as the cornerstone of spine, the body always tried to maintain the whole spino-pelvic balance through the interaction of various parts of the mechanical chain of the spine and pelvis. In the past, many studies focused on the regulation of the distal pelvis of the lumbar spine, and the compensation mechanism of the proximal end has been gradually quantified in recent years. In this study, the sagittal plane parameters of these kinds of patients were statistically analyzed, and it was concluded that the sagittal plane balance was regulated and maintained by LL, PT, and TK.

Regarding the sagittal compensatory mechanism of DK, Barrey et al. (2013) think that the severity of the imbalance corresponds to three stages: the state of equilibrium, the balance under the compensatory mechanism, and the state of imbalance. The influence of the loss of thoracolumbar and lumbar curvature overall sagittal sequence is compensated and helps to maintain sagittal balance. Once the deformity aggravates and the compensation mechanism cannot maintain balance, the state of decompensation will occur. According to the sagittal parameters of the patient, Schwab et al. (2014) proposed that the patient’s PT is greater than 20° and the patient’s pelvic rotation is in the limit state, which cannot be compensated effectively, and the patient is in a state of decompensation. Clinically, the characteristic of the DK was the loss of LL and/or the increase of TLK (Roussouly and Pinheiro-Franco, 2011). Knowing the relationship between sagittal plane parameters in patients with DK was of great importance, which could better evaluate the patient and guide surgical treatment (Le Huec et al., 2015b). Therefore, in this paper, we divided the DK patients into two groups according to different PT: balance group and imbalance group, and explored the relationship between different sagittal parameters in different groups.

In our study, in the balance group, the LL was correlated with TK. The formula was LL = 33.57 + 0.33 × TK. For the DK patients with balance, the sagittal alignment was in a compensatory state. Due to the degeneration of the spine, the LL decreased. To keep the balance, the back-muscle system and skeleton system cooperated with each other as a whole, which makes the body stay in a balanced state (Liu et al., 2020). With the decrease of the LL, the pelvic retroversion and the TK decreased, which make the spine balanced. Through this study, we can see that the LL was correlated with TK but not PT. Because for such patients, the pelvis was in an adjustable state, the spine starts the self-adjustment mechanism. The reduction of the TK was to accommodate the decrease of LL, which can make the patient stay balanced enough (Lamartina et al., 2012; Bassani et al., 2019). Besides, the formula could provide a guideline of the surgical treatment. The main purpose of the operation was to restore the LL to alleviate the back pain in the treatment of the balanced DK patients. Over restoration of the LL may result in the enlargement of TK, which contributes to the high incidence of proximal junction kyphosis (PJK), so the LL should be properly restored to avoid the complication.

For the imbalance group, the LL was correlated with TK and PT, the formula was LL = 18.2−0.26 × PT + 0.62 × TK. For such patients, the body was out of control for various reasons. The reduction of the TK was not enough to make the patient stay in balance and the pelvic adjustment mechanism activated. Both try their best to put the whole body in an upright position. The patient’s pelvic rotation is in a limited state, which cannot be compensated effectively (Le Huec et al., 2011; Lee et al., 2011). Therefore, for such patients, restoring the LL was not enough, and correcting the pelvic rotation was also necessary. The methods of the correction of pelvic rotation should be used in the treatment of such patients.

The current research has several limitations. Firstly, it is a retrospective study, which raises a concern about selection bias and loss of follow-up. Secondly, we only concentrated on the sagittal alignment of the patients, and coronal balance should be further studied in the next framework. Eventually, the number of cases in our study was relatively small. Larger case studies should be conducted in the future.

## Conclusion

For the patients with DK, the sagittal balance was adjusted by different sagittal parameters, which positions the body. From the present study, we can see that the LL has a significant correlation with PT and TK in patients with DK, especially in the SIB group. So, the LL and pelvic rotation should both be of concern during the correction. While in SB, the LL was only correlated with TK. Over restoration of the LL may contribute to a high incidence of PJK. Therefore, the correction of LL in different groups should be calculated to avoid complication.

## Data Availability Statement

The original contributions presented in the study are included in the article/supplementary material, further inquiries can be directed to the corresponding authors.

## Ethics Statement

The protocol was reviewed and approved by the Ethics committee of Peking University of people’s hospital. Written informed consent was obtained from all participants for their participation in this study.

## Author Contributions

HL and YL: conceptualization. HL, SX, and CG: data curation. YL and SX: formal analysis and software. YL and CG: investigation. SX, YL, and KM: methodology. HL: project administration. SX and KM: resources. YL: validation. HL: visualization. HL, SX, and YL: writing and editing. All authors contributed to the article and approved the submitted version.

## Conflict of Interest

The authors declare that the research was conducted in the absence of any commercial or financial relationships that could be construed as a potential conflict of interest.

## Publisher’s Note

All claims expressed in this article are solely those of the authors and do not necessarily represent those of their affiliated organizations, or those of the publisher, the editors and the reviewers. Any product that may be evaluated in this article, or claim that may be made by its manufacturer, is not guaranteed or endorsed by the publisher.

## Abbreviations

DK: degenerative kyphosis
DTLK: degenerative thoracolumbar kyphosis
DLLL: loss of lumbar lordosis
PI: pelvic incidence
PT: pelvic tilt
SB: sagittal balance
SIB: sagittal imbalance
SVA: sagittal vertical axis.
